# Management of 3 Avulsed Permanent Teeth: Case Report of a 3-Year Follow-up

**DOI:** 10.1155/2022/2081684

**Published:** 2022-03-16

**Authors:** Soukaina El Kharroubi, Sofia Drouri, Bouchra Doumari, Sara Dhoum, Hafsa El Merini

**Affiliations:** Department of Conservative Dentistry and Endodontics, School of Dentistry, Hassan II University of Casablanca, Morocco

## Abstract

Dental avulsion is one of the most serious dental injuries. When the tooth is knocked out, the blood supply to the pulp is interrupted and the periodontal ligament cells are exposed to injuries caused by the external environment. In recent years, research findings have shown the possibility of complete healing under favorable conditions: replanting the tooth according to the criteria required by the guideline such as the extra oral time which should be very short, the time of pulp removal after replantation, and the appropriate storage medium. This set of criteria may lead to a slower progression of the phenomenon of resorption with a better prognosis over time; however, there is a high risk of complications such as external root resorption which lies in late replantation. The research results of many research-groups around the world have given us a better understanding of healing and complications. Nowadays, even teeth that are replanted after a delay can be saved. The dentist remains the key person in the case of dental trauma, namely, dental avulsion as his role is the management of the emergency situations. The objective of this case study is to present a successful management of 3 avulsed permanent incisors, replanted and remained with no complications for over a period of 3 years.

## 1. Introduction

Tooth avulsion is a serious traumatic dental injury (total luxation, extrusion, or avulsion). It is a dental trauma that corresponds to the complete displacement of the tooth out of the alveolar bone socket [1–3]. Avulsion of permanent teeth varies from 0.5% to 16% of all traumatic injuries, as it is one of the rare and serious dental injuries. The young population is the most affected and the maxillary central incisors are the teeth most often involved due to their exposed position in the dental arch [4]. The risk of infection and root resorption can occur at any time after avulsion and dental replantation, which may influence the treatment outcome and survival rate including the prognosis of the teeth involved [5]. Besides, the consequences of dental avulsion are countless; they differ from patient to patient and may be related to the individuals' quality of life, psychological, and social problems, as well as the costs of treatment. [6]. In case of tooth avulsion, most periodontal ligament cells left on the root surface are due to the tearing of the alveolar-dental ligament; these cells must be hydrated to maintain the durability of the tooth while reducing the phenomenon of resorption and allowing healing. However, when the extraoral time increases, leaving the periodontal ligament dry, inflammatory resorption occurs [4]. The incidence of an external root resorption (inflammatory/replacement) remains high even in a correct management of replantation of the tooth. However, several criteria can lead to slow progression and a better prognosis over time if they are well respected. [6] After all, the natural history of inflammatory and replacement resorption is important in order to detect the right prognosis of the replanted tooth. [6]

The aim of this study/case report is to present a successful replantation of 3 avulsed permanent maxillary incisors. No sign of resorption and ankylosis were seen over a period of 3 years.

## 2. Case Report

**Figure 1:**
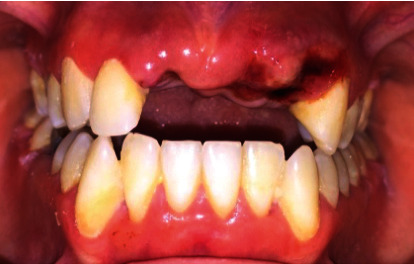
Intraoral view showing avulsion of teeth 11, 21, and 22.

**Figure 2:**
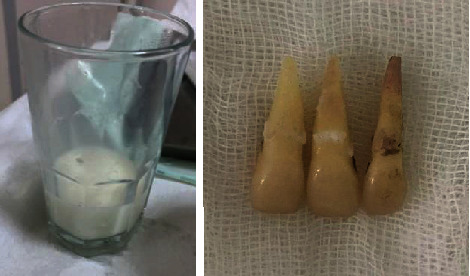
Avulsed teeth conserved in milk for 60 minutes—intact teeth.

A 28-year-old female patient was referred to the Department of Conservative Dentistry and Endodontics at the School of Dentistry (Casablanca-Morocco) after an assault causing three avulsions of maxillary incisors after one hour of trauma. The patient was examined for extraoral signs of injury, including swelling and asymmetry of the face. No other oral injury was clinically detected. The patient reported that she has a history of periodontitis already treated and stabilized. The intraoral examination revealed that the maxillary permanent central incisors and lateral left incisor (tooth 11, 21, and 22) were avulsed with lacerations of soft tissue of gingiva (Figure 1). The avulsed teeth had been kept in whole milk, from the moment of trauma until emergency visit 60 minutes later. The crowns of the avulsed teeth were intact, and the roots had closed apexes (Figure 2).

**Figure 3:**
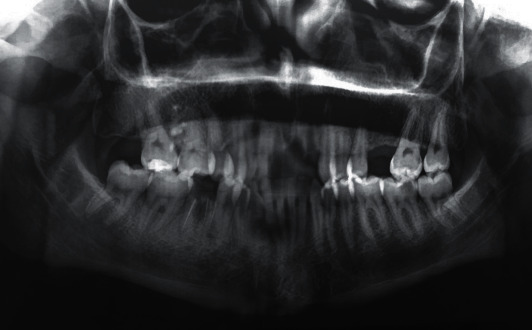
Preoperative panoramic radiograph showing empty sockets of 11, 21, and 22. No sign of fracture or contusion of the alveolar sockets.

**Figure 4:**
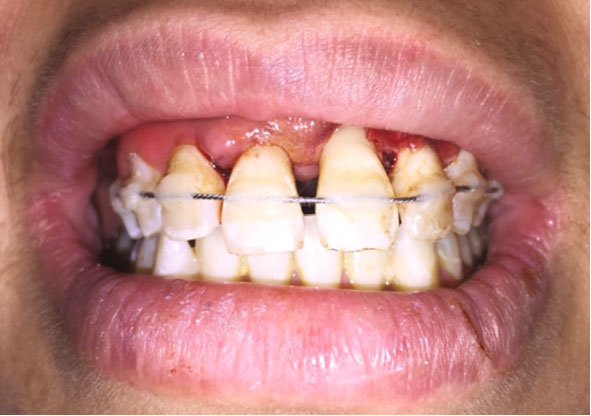
Immediate replanted and splinted teeth 11, 21, and 22. A flexible splint stabilized teeth for 4 weeks.

Preoperative panoramic radiograph showed empty sockets of 11, 21, and 22 (Figure 3) The alveolar bone losses resulting from periodontal disease on these teeth and other teeth are also observed in this radiograph. The roots and sockets were cleaned gently with a saline rinse and the teeth replanted manually compressed to its original position under local anesthesia. Placement of teeth was confirmed radiographically before a flexible splint was placed for 4 weeks (Figure 4). Systemic antibiotic therapy was prescribed (penicillin 1 million units immediately, thereafter 2 million units daily for 6 days). A soft diet, good oral hygiene with the use of a soft- bristled toothbrush, and a 0.12% chlorhexidine mouth rinse were also prescribed for 2 weeks.

**Figure 5:**
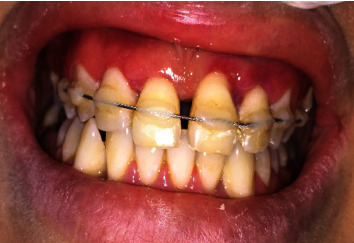
1-week posttrauma.

**Figure 6:**
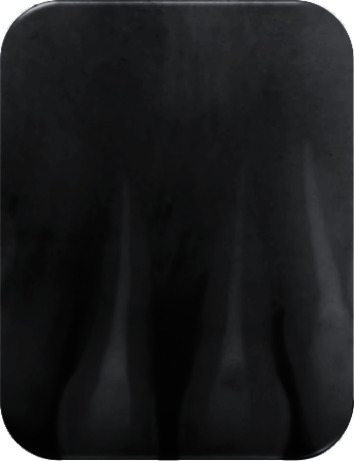
Obturation canals after 3-months medication with replanted calcium hydroxide treatment.

**Figure 7:**
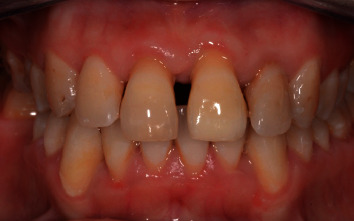
4-week posttrauma.

**Figure 8:**
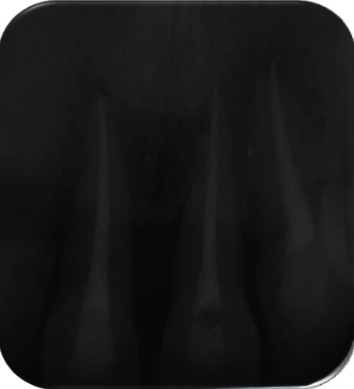
18-month follow-up of teeth.

**Figure 9:**
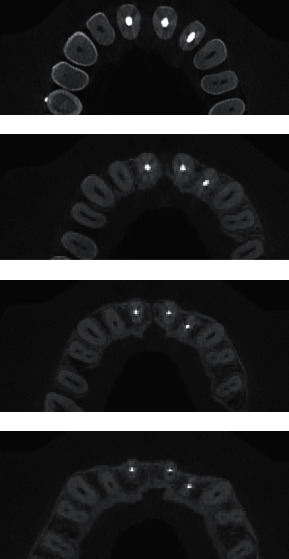
CBCT images—3 years after replantation.

**Figure 10:**
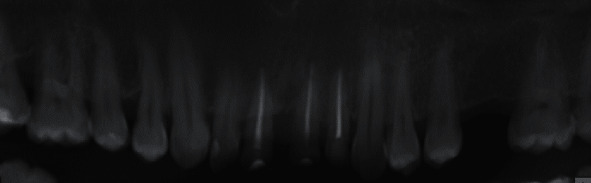
No pathology and resorption. 3-year radiograph examination.

The patient was called after one-week postreplantation (Figure 5) for an intracanal placement of calcium hydroxide which was renewed for after 3 months, and the canals were obturated with Gutta-percha later (Figure 6). The splinting wire was removed after 4 weeks (Figure 7). Successive radiograph testing (periapical and CBCT) was performed in the follow-up period to disclose root resorption (Figures 8, 9, and 10) The radiographic tests showed no evidence of root resorption. A periodontal bone loss has been detected with absence of the gingival inflammation or bleeding on probing (absence of periodontal pocket); this represents a reliable indicator of periodontal stability.

## 3. Discussion

Dental avulsion mostly affects children and adolescents with a prevalence of 17.5%. It is mostly found among males than females [6].

### 3.1. The Different Criteria Affecting the Management of the Replanted Avulsed Teeth

The incidence of the external root resorption (inflammatory/replacement) can be high and varies between 59% and 80% in patients with so-called correct replantation [6]. Several studies have investigated the effect of dry storage that plays an important role in the viability of the periodontal ligament cells and may be responsible for the occurrence of inflammatory resorption. It has also been shown that the periodontal ligament cells can survive a drying time of 10 to 15 minutes, but the possibility of their survival beyond this time where the drying time exceeds 60 minutes may be very limited; as a result, the risk of early resorption increases [7]. Therefore, milk remains the correct balanced storage medium. It is the most relevant medium since it preserves the cells of the periodontal ligament for several hours and keeps them in a good condition until replantation. A balanced salt and saliva solution could also have the same indications effects as milk [8]. As in this present case, the avulsed teeth were kept in milk for 60 minutes from the moment of trauma until emergency visit and remained intact. The factors associated with avulsion injury show that an immature tooth develop more complications and have a lower survival rate than a mature tooth [9]. In this present clinical case, this factor is favorable, with three teeth closed apexes. Another study shows that the age of the patient can be a good factor to determine the progression of root resorption in teeth with an extended extra oral time. [6] Rightly as well, patients between 17 and 39 years old present higher rates at the time of the avulsion trauma in comparison to younger patients between 8 and 16 years old.

A recent study also shows that the risk of developing severe inflammatory resorption is related to the pulpectomy time. The timing of pulp extirpation after replantation is very important and it must be done promptly to reduce the risk of early complications [10]. Regarding our case, the pulpectomy was done after one-week postreplantation and the intracanal calcium hydroxide was placed for 3 months before the obturation canals. This aspect of the treatment of replanted teeth/a replanted tooth has been the most controversial and has undergone several changes over time. In the past clinical and experimental studies, endodontic treatment (biomechanical preparation followed by obturation with Gutta-percha and sealer) used to be performed extraorally before the replantation. [11] Nowadays, guidelines recommend endodontic treatment to be performed intraorally because it can minimize the extraoral time and reduce many risk factors [12]. As in our case, the canals' obturation with Gutta-percha and sealer was performed intraorally. From a previously reported clinical study of replanted teeth [13], the increase of the duration of the dry time can lead to the increase of the risk of ankylosis which is significantly higher with mature teeth that have closed apex than with immature teeth [13] [14]. Moreover, it has been shown that internal and external root resorption (inflammatory or replacement) have different incidences after dental avulsion and replantation [5]. As a result, it was found that the replacement root resorption is the most prominent, followed by the inflammatory external root resorption, then the surface root resorption, and finally, the internal root resorption which is the least common. Periodontal disease has been considered a contraindication to replantation. However, various studies have shown that periodontitis is no more an absolute contraindication to dental replantation [15]. Teeth with one periodontal pocket >6 mm in patients under the age of 40 had a 2.5- and 2.6-fold lower risk of failure, compared to the teeth with more pockets and patients over the age of 40. These factors should be carefully observed for teeth replantation [15]. Other studies have shown that delayed replantation does not fully maintain the bone volume as the bone disappears with the tooth. In the anterior region of the maxilla, the buccal profile of the alveolar bone depends on teeth with a feasible periodontal ligament [16]. In our case study, the conditions for the replantation of the 3 teeth were favorable as the young patient had stable periodontitis that was successfully treated, and the maintenance phase of periodontal therapy performed since that time. The absence of inflammation and bleeding on probing can be used as predictors of the periodontal stability.

### 3.2. Prognosis

The prognosis of an avulsed replanted permanent tooth depends on different criteria as the amount of damage to the root surface, the degree of the root development of the avulsed teeth, and the extra-alveolar dry period and storage medium in which the tooth was kept prior to replantation. There are many factors that can affect the clinical success of a tooth replantation such as endodontic treatment, antibiotics prescription, age of the patient, type of the splinting used, and the time of the replantation, as well as the time kept for the storage [3]. There are reports of replanted teeth having favorable prognosis and could last for over 30 years. Some of them remain in function for 5 years or more, but most of them are lost due to root resorption or other complications like ankylosis. However, a good management of the infection may lead to a tissue regeneration and give a good environment for its healing [17]. It has been reported that a favorable prognosis of a replanted teeth with an arrested external root resorption after a 2-year follow-up has shown that the use of milk as a storage medium maintain the viability of periodontal ligament cells [18]. As for our case, the 3 replanted teeth have not shown any sign of root resorption or ankylosis for 3 years. The minimum extraoral time and the appropriate storage medium after avulsion are directly associated with good prognosis of a replanted tooth staying on the dental arch for a longtime. This may happen because of a minimal infection, maintenance of root cementum, and a periodontal ligament cell on root surface remained alive. [19]

Below are the different factors influencing the prognosis of the avulsed tooth [20]:

- The type and the timing of the treatment
- Pulp removal (root canal treatment) after tooth replantation within 7-14 days showed a significantly higher survival rate
- Contraindications to replantation: uncooperative patient, many severe caries, advanced and untreated periodontal diseases, and serious general pathology (immunosuppression, severe cardiac conditions)
- Immature root (open apex) exhibits more complications and usually has worse prognosis compared to mature root (closed apex) with a lower survival rate
- Appropriate storage media increase the success rate of replantation
- better treatment outcome lies in the immediate replantation of the avulsed tooth
- The prognosis of replanted teeth is determined by the patient's compliance (postreplantation management and follow-up appointments).

Therefore, it can be concluded that tooth avulsion remains a severe dental injury with an unpredictable prognosis.

## 4. Conclusion

Although tooth avulsion is one of the most complicated types of teeth traumas. We were able to maintain a 3-year follow-up of the 3 replanted teeth with no sign of root resorption or ankylosis. Our treatment can be considered a successful one with a favorable prognosis.

A regular clinical and radiographic examination follow-up every year is fundamental.
